# Evaluating Data Abstraction Assistant, a novel software application for data abstraction during systematic reviews: protocol for a randomized controlled trial

**DOI:** 10.1186/s13643-016-0373-7

**Published:** 2016-11-22

**Authors:** Ian J. Saldanha, Christopher H. Schmid, Joseph Lau, Kay Dickersin, Jesse A. Berlin, Jens Jap, Bryant T. Smith, Simona Carini, Wiley Chan, Berry De Bruijn, Byron C. Wallace, Susan M. Hutfless, Ida Sim, M. Hassan Murad, Sandra A. Walsh, Elizabeth J. Whamond, Tianjing Li

**Affiliations:** 1 Department of Epidemiology, Johns Hopkins Bloomberg School of Public Health, 615 North Wolfe Street, Room W6507-B, Baltimore, MD 21205 USA; 2 Department of Biostatistics, and Center for Evidence-based Medicine, Brown University School of Public Health, Providence, RI USA; 3 Department of Health Services, Policy and Practice, and Center for Evidence-based Medicine, Brown University School of Public Health, Providence, RI USA; 4 Epidemiology, Johnson & Johnson, Titusville, NJ USA; 5 Center for Evidence-based Medicine, Brown University School of Public Health, Providence, RI USA; 6 Department of Medicine, University of California San Francisco School of Medicine, San Francisco, CA USA; 7 Internal Medicine, Kaiser Permanente Northwest, Portland, OR USA; 8 National Research Council Information and Communications Technologies Portfolio (NRC-ICT), Ottawa, ON Canada; 9 Northeastern University College of Computer and Information Science, Boston, MA USA; 10 Department of Medicine, Johns Hopkins School of Medicine, Baltimore, MD USA; 11 Department of Medicine, University of California San Francisco School of Medicine, San Francisco, CA USA; 12 College of Medicine, and Evidence-based Practice Center, Mayo Clinic, ᅟRochester, MN USA; 13 California Breast Cancer Organizations, Davis, CA USA; 14 Cochrane Consumer Network, Fredericton, NB Canada

**Keywords:** Data abstraction, Systematic reviews, Randomized controlled trial

## Abstract

**Background:**

Data abstraction, a critical systematic review step, is time-consuming and prone to errors. Current standards for approaches to data abstraction rest on a weak evidence base. We developed the Data Abstraction Assistant (DAA), a novel software application designed to facilitate the abstraction process by allowing users to (1) view study article PDFs juxtaposed to electronic data abstraction forms linked to a data abstraction system, (2) highlight (or “pin”) the location of the text in the PDF, and (3) copy relevant text from the PDF into the form. We describe the design of a randomized controlled trial (RCT) that compares the relative effectiveness of (A) DAA-facilitated single abstraction plus verification by a second person, (B) traditional (non-DAA-facilitated) single abstraction plus verification by a second person, and (C) traditional independent dual abstraction plus adjudication to ascertain the accuracy and efficiency of abstraction.

**Methods:**

This is an online, randomized, three-arm, crossover trial. We will enroll 24 pairs of abstractors (i.e., sample size is 48 participants), each pair comprising one less and one more experienced abstractor. Pairs will be randomized to abstract data from six articles, two under each of the three approaches. Abstractors will complete pre-tested data abstraction forms using the Systematic Review Data Repository (SRDR), an online data abstraction system. The primary outcomes are (1) proportion of data items abstracted that constitute an error (compared with an answer key) and (2) total time taken to complete abstraction (by two abstractors in the pair, including verification and/or adjudication).

**Discussion:**

The DAA trial uses a practical design to test a novel software application as a tool to help improve the accuracy and efficiency of the data abstraction process during systematic reviews. Findings from the DAA trial will provide much-needed evidence to strengthen current recommendations for data abstraction approaches.

**Trial registration:**

The trial is registered at National Information Center on Health Services Research and Health Care Technology (NICHSR) under Registration # HSRP20152269: https://wwwcf.nlm.nih.gov/hsr_project/view_hsrproj_record.cfm?NLMUNIQUE_ID=20152269&SEARCH_FOR=Tianjing%20Li. All items from the World Health Organization Trial Registration Data Set are covered at various locations in this protocol. Protocol version and date: This is version 2.0 of the protocol, dated September 6, 2016. As needed, we will communicate any protocol amendments to the Institutional Review Boards (IRBs) of Johns Hopkins Bloomberg School of Public Health (JHBSPH) and Brown University. We also will make appropriate as-needed modifications to the NICHSR website in a timely fashion.

**Electronic supplementary material:**

The online version of this article (doi:10.1186/s13643-016-0373-7) contains supplementary material, which is available to authorized users.

## Background

Systematic reviews (“reviews”) are comparative effectiveness research studies that use explicit methods to identify, appraise, and synthesize the research evidence addressing a given research question [1]. The steps in completing a review include formulating the research question, finding and collecting data from individual studies, and synthesizing the evidence [2]. The validity of the review findings is contingent upon accurate and complete data collection from journal articles reporting results of relevant studies (“articles”), a process known as *data abstraction* (also known as *data extraction*).

As a predominantly manual process, data abstraction is inefficient, being both labor-intensive and error-prone. Errors during abstraction are common and have been well documented in the literature [3–6]. One study estimated that the error rate, defined as “any small discrepancy from the reference standard,” was approximately 30% for single abstraction, regardless of the level of abstractor experience [6]. Our pilot data showed that less experienced abstractors made more errors across all types of research questions, and errors were highest for numerical results [7]. Abstraction errors occur when abstractors either omit from the abstraction information that is present in the article or when information is abstracted incorrectly. When Gøtzsche and colleagues examined 27 meta-analyses (i.e., statistical analyses of results from included studies in systematic reviews) published in 2004 across a range of topics, they found multiple errors in 37% of meta-analyses [4]. In another study, Jones and colleagues documented abstraction errors in 20/42 reviews (48%); in all cases, the errors changed the summary meta-analytic results, although none changed the review conclusions [5].

Currently recommended approaches to reducing errors in data abstraction fall into two categories: (1) abstraction by one person followed by checking of the abstraction by a second person (“single abstraction plus verification”) and (2) independent abstraction by two persons followed by resolution of any discrepancies (“independent dual abstraction plus adjudication”). The former approach also sometimes concludes with adjudication between the two abstractors. Buscemi and colleagues showed that single abstraction plus verification results in approximately 20% more errors than independent dual abstraction plus adjudication, but the latter approach takes approximately 50% longer [3].

Because only one study [3] has examined the tradeoffs between single abstraction plus verification and independent dual abstraction plus adjudication and that study focused on a single review topic with only four abstractors, current standards for data abstraction rest on a weak evidence base. Major sponsors and producers of reviews (e.g., Agency for Healthcare Research and Quality Evidence-based Practice Centers (AHRQ EPCs), Cochrane, Centre for Research and Dissemination (CRD)) and organizations that develop methodology standards for reviews (e.g., AHRQ, Cochrane, Institute of Medicine (IOM)) are inconsistent in their recommendations for approaches to reduce errors in abstraction [1, 2, 8–10]. Because “*so little is known about how best to optimize accuracy and efficiency*” [1], the IOM Committee stopped short of recommending independent dual abstraction for all data elements. Instead, the IOM recommended: “*at minimum, use two or more researchers, working independently, to extract quantitative and other critical data from each study*” [1]*.* Thus, although the IOM recommended independent dual abstraction for “critical data,” an important gap in our current methodological understanding of data abstraction remains. The recommendation for “critical data” could represent unnecessary work or, conversely, the IOM’s implicit recommendation that a single person could abstract non-critical data could represent an opportunity for error.

Computer-aided abstraction could potentially make the abstraction process more efficient and more accurate by facilitating the location and storage of key information in articles. With funding from the Patient Centered Outcomes Research Institute (PCORI), we developed the Data Abstraction Assistant (DAA), a novel software application designed to facilitate tracking the location of abstracted information in articles and to reduce errors during abstraction. DAA facilitates abstraction by allowing users to (1) view article PDFs juxtaposed to electronic data abstraction forms in data abstraction systems, (2) highlight (or “pin”) the location of text in the PDF, and (3) copy text automatically from the PDF into the form.

We are conducting a randomized controlled trial (RCT) to compare the relative effectiveness of (A) DAA-facilitated single abstraction plus verification, (B) traditional (non-DAA-facilitated) single abstraction plus verification, and (C) traditional independent dual abstraction plus adjudication on the accuracy and efficiency of abstraction. The objective of this manuscript is to describe the design of our RCT adhering to the Standard Protocol Items: Recommendations for Interventional Trials (SPIRIT) evidence-based guideline for reporting protocols of RCTs [11] (Additional file 1). Our searches of PubMed and *The Cochrane Library* up to August 26, 2016, did not identify any RCT or systematic review of RCTs that have compared the accuracy and efficiency of various data abstraction approaches.

## Methods

### Study design and setting

**Fig. 1:**
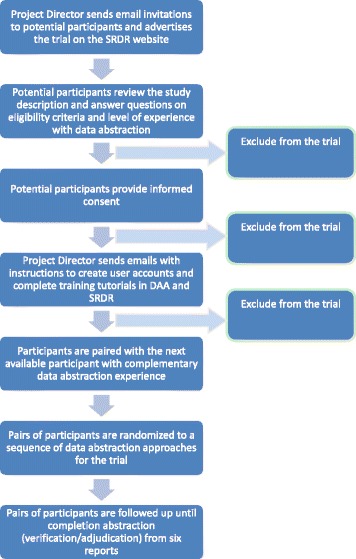
Flow of participants during the trial

We have designed this study as a randomized, three-arm, crossover trial. The trial flowchart is presented in Fig. 1. This trial will be conducted entirely online. However, during verification of abstracted data or adjudication of discrepancies, participants (abstractors) in a pair will have the option to communicate with each other using any preferred mode of communication (e.g., video call, phone call, in-person meeting).

### Study population

The intended study population is individuals, including researchers, patients, clinicians, and methodologists, who have previously participated in data abstraction for systematic reviews, without restriction on the number, types, or topics of reviews.

### Eligibility criteria

The trial will only include individuals who meet all the following criteria:

- At least 20 years of age
- Self-reported proficiency with reading scientific articles in English
- Completed abstraction for at least one journal article for a systematic review in any field
- Provided informed consent

### Recruitment

For this trial, we will use the Systematic Review Data Repository (SRDR) as the data abstraction system. SRDR, maintained by the Brown University EPC (http://srdr.ahrq.gov), is state-of-the-art, open-source, open-access, and available free of charge to anyone conducting a review [12, 13]. We will utilize four strategies to identify and recruit potential abstractors: (1) emails to students who have registered for at least one course in systematic review methods through Johns Hopkins Bloomberg School of Public Health (JHBSPH) and Brown University; (2) emails to faculty and staff at Johns Hopkins and Brown EPCs; (3) advertising on the SRDR website; and (4) advertising through patient organizations such as Consumers United for Evidence-based Healthcare (CUE) and Cochrane Consumer Network (CCNet). CUE is a US-based coalition of health and patient advocacy organizations committed to empowering patients to make the best use of evidence-based healthcare (http://us.cochrane.org/CUE). CCNet’s primary role is to get patients around the world involved in the production of Cochrane reviews (http://consumers.cochrane.org). All potentially eligible abstractors are directed to the DAA Trial Data Abstractor Enrollment website (“Enrollment website”), the informational page for the trial (http://srdr.ahrq.gov/daa/info).

### Participant enrollment, training, and pairing

To mimic how individuals are often paired for data abstraction in real-world reviews, we will form pairs of abstractors consisting of one less experienced abstractor and one more experienced abstractor. The Enrollment website will ask participants to answer questions related to their eligibility and level of experience with abstraction for reviews. Based on the results of a pilot study (Additional file 2), we determined that the number of published reviews authored, dichotomized at fewer than 3 versus 3 or more, was best able to classify abstractors as “less” or “more” experienced with abstraction, respectively.

Once an abstractor is deemed to have met all eligibility criteria for the trial and has provided information regarding experience with abstraction, the Enrollment website will notify the abstractor that s/he is eligible for participation and, upon the click of a button, will direct the participant to the DAA Trial Informed Consent website (“Consent website”) (http://srdr.ahrq.gov/daa/consent). The Consent website will automatically notify the Project Director regarding the names and email addresses of abstractors who have successfully provided informed consent.

Abstractors will be required to complete training in using SRDR and DAA before being paired and randomized. Once an abstractor has completed training, the Project Director pairs the abstractor with the next available abstractor, in chronological order, who has complementary abstraction experience (i.e., each pair will include one less experienced and one more experienced abstractor).

### Randomization of pairs of abstractors

Abstractors will be randomized as pairs. Each pair will complete abstraction for six articles, two under each of the three approaches (A = DAA-facilitated single abstraction plus verification; B = traditional [non-DAA-facilitated] single abstraction plus verification; and C = traditional independent dual abstraction plus adjudication). This is to reduce possible contamination from the learning process and for ease of coordination. To maximize efficiency, we will use a crossover design such that each pair of abstractors will implement all three approaches being evaluated, with the intent of estimating differences within pairs. The six possible sequences are AABBCC, AACCBB, BBCCAA, BBAACC, CCAABB, and CCBBAA. Table 1 lays out the assignment of 24 abstractor pairs to sequences (*n* = 6) and to reviews (*n* = 4). For further explanation of sample size calculation and review selection, see sections titled “Sample size and power calculation” and “Identification of studies and outcomes for abstraction during the trial”, respectively. We will randomly assign each consecutive pair of abstractors to a “slot” (row in Table 1). The Senior Statistician will use the R statistical environment to generate the random order from 1 to 24. For example, if the first random number is 17, the first pair will be assigned to “pair 17”, and will abstract data from articles 31 to 36 according to sequence BBAACC, as shown in Table 1.

**Table 1:** Assignment of 24 pairs of abstractors to 6 sequences and to 48 articles

								Random sequence
	Article 1	Article 2	Article 3	Article 4	Article 5	Article 6	Articles selected from systematic review #1	
Pair 1	A	A	B	B	C	C		Sequence 1
Pair 2	B	B	C	C	A	A		Sequence 2
Pair 3	C	C	A	A	B	B		Sequence 3
	Article 7	Article 8	Article 9	Article 10	Article 11	Article 12		
Pair 4	A	A	C	C	B	B		Sequence 4
Pair 5	B	B	A	A	C	C		Sequence 5
Pair 6	C	C	B	B	A	A		Sequence 6
	Article 13	Article 14	Article 15	Article 16	Article 17	Article 18	Articles selected from systematic review #2	
Pair 7	A	A	B	B	C	C		Sequence 1
Pair 8	B	B	C	C	A	A		Sequence 2
Pair 9	C	C	A	A	B	B		Sequence 3
	Article 19	Article 20	Article 21	Article 22	Article 23	Article 24		
Pair 10	A	A	C	C	B	B		Sequence 4
Pair 11	B	B	A	A	C	C		Sequence 5
Pair 12	C	C	B	B	A	A		Sequence 6
	Article 25	Article 26	Article 27	Article 28	Article 29	Article 30	Articles selected from systematic review #3	
Pair 13	A	A	B	B	C	C		Sequence 1
Pair 14	B	B	C	C	A	A		Sequence 2
Pair 15	C	C	A	A	B	B		Sequence 3
	Article 31	Article 32	Article 33	Article 34	Article 35	Article 36		
Pair 16	A	A	C	C	B	B		Sequence 4
Pair 17	B	B	A	A	C	C		Sequence 5
Pair 18	C	C	B	B	A	A		Sequence 6
	Article 37	Article 38	Article 39	Article 40	Article 41	Article 42	Articles selected from systematic review #4	
Pair 19	A	A	B	B	C	C		Sequence 1
Pair 20	B	B	C	C	A	A		Sequence 2
Pair 21	C	C	A	A	B	B		Sequence 3
	Article 43	Article 44	Article 45	Article 46	Article 47	Article 48		
Pair 22	A	A	C	C	B	B		Sequence 4
Pair 23	B	B	A	A	C	C		Sequence 5
Pair 24	C	C	B	B	A	A		Sequence 6

A, B, and C denote three different approaches for data abstraction; see the section “Study arms (Abstraction approaches)”. Random sequence is the permuted arrangement of three approaches for data abstraction. For example, sequence 1 indicates data abstractors will collect data from 6 unique articles using AABBCC approaches respectively

The Project Director will release two articles at a time. To maintain allocation concealment, we will keep the Project Director, who is responsible for pairing abstractors and communicating the randomized sequence to the pair, unaware of the next slot assigned. For this reason, the Senior Statistician alone will have access to the random order. For each pair to be randomized, the Project Director will contact and receive from the Senior Statistician the randomized slot to which the pair will be assigned.

### Study arms (abstraction approaches)

#### Approach A—DAA-facilitated single abstraction plus verification

In Approach A, which uses DAA, the less experienced abstractor in a pair will complete the abstraction form first, followed by the more experienced abstractor who will verify the information abstracted by her/his less experienced partner. The less experienced abstractor will complete abstraction for the two assigned articles using DAA and the abstraction form in SRDR by placing a pin identifying each location of the PDF text supporting the answer to every question on the abstraction form. The software allows multiple locations of the PDF text to be pinned for a given question. Once the initial abstraction is completed, the more experienced abstractor will be given access the abstracted data for the two articles in SRDR, together with the pinned locations on the PDFs. The more experienced abstractor can change any of the less experienced abstractor’s responses as s/he considers appropriate (verification) and, if desired, request discussion with the less experienced abstractor (data adjudication). Once the more experienced abstractor has verified the data abstracted for an article (with or without discussion with the less experienced abstractor), abstraction for that article will be considered complete.

#### Approach B—Traditional single abstraction plus verification

Approach B does *not* use DAA. As in Approach A, the less experienced abstractor in a pair will complete the abstraction form first (without using DAA), followed by the more experienced abstractor who will verify the information abstracted by her/his less experienced partner. The less experienced abstractor in a pair will complete abstraction for the assigned articles using the abstraction form in SRDR. Once the abstraction by the less experienced abstractor is completed, the more experienced abstractor will be given access the abstracted data for the two articles in SRDR. The more experienced abstractor can change any of the less experienced abstractor’s responses as s/he considers appropriate (verification) and, if desired, request discussion with the less experienced abstractor to adjudicate the data (data adjudication). As in Approach A, once the more experienced abstractor has verified the abstracted data for an article (with or without discussion with the less experienced abstractor), abstraction for that article is considered complete.

#### Approach C—Traditional independent dual abstraction plus adjudication

Approach C, which also does *not* use DAA, involves two main steps. In the first step, the two abstractors in a pair each will abstract data independently for the two assigned articles using the abstraction form in SRDR. The two abstractors will inform each other that they have completed their independent abstractions and will develop a plan for adjudication (e.g., video call, phone call, in-person meeting). In the second step, the abstractors will compare their abstractions and address any discrepancies in the abstracted data for the two articles (data adjudication). Once the two abstractors arrive at consensus on all abstracted data for a given article, abstraction for that article is considered complete. As appropriate, both abstractors will edit their own incorrect answers. We will not allow a third abstractor to resolve discrepancies.

### Masking

It is not feasible to mask abstractors or the Project Director because the abstractors need to be aware of the abstraction approach in order to abstract data, and the Project Director needs to be aware of the sequence of assigned approaches to allocate articles and follow abstractors through the trial. The data analysts will use computer programs, such as the R statistical environment, to detect errors (a step that does not involve subjective judgment), and will not be masked.

### Follow-up and retention of participants

To maximize retention, the Project Director will maintain regular email contact with each abstractor (or pair of abstractors, as appropriate) throughout the trial. Following are the scheduled junctures for email contact: after screening and consent, once DAA and SRDR trainings are completed, after randomization, and after abstraction under each approach (two articles) is completed. Abstractors will be followed throughout the trial unless consent is withdrawn. In instances where an abstractor or pair of abstractors does not complete abstraction for the assigned articles, we will make every effort to encourage completion of abstraction. If abstraction is not completed after five weekly email reminders, or if consent is withdrawn, we will replace the abstractor with a previously not enrolled abstractor with the same level of abstraction experience, or replace both abstractors in the pair, as needed, so that the remaining abstraction is completed.

We will provide each abstractor US $250 as compensation for participation in the trial. Compensation will be provided only once abstraction for all six articles has been completed (i.e., no partial/interim payment).

### Identification of studies and outcomes for abstraction during the trial

We have identified 48 journal articles from four reviews reporting results of RCTs (12 articles per review), and these are the articles that will be abstracted from during the trial. We identified the reviews by searching MEDLINE and the Cochrane Database of Systematic Reviews for a range of clinical topic areas. To ensure that the reviews and the outcomes are relevant to patients, our consumer co-investigators (SAW, EJW, and Ms. Vernal Branch) were involved in the selection of reviews and outcomes. In cases where a review includes more than 12 articles, we selected 12 articles that reported the most number of outcomes. We have included one article for each trial (i.e., not multiple publications, conference abstracts, or data from trial registries).

The reviews chosen are (1) multi-factorial interventions to prevent falls in older adults [14]; (2) proprotein convertase subtilisin/kexin type 9 (PCSK-9) antibodies for adults with hypercholesterolemia [15]; (3) interventions to promote physical activity in cancer survivors [16]; and (4) omega-3 fatty acids for adults with depression [17].

### Data collection, management, and monitoring

All data during the trial will be collected via websites, SRDR (the data abstraction system), and DAA (the document management system). We have developed and pilot tested a separate data abstraction form compatible with SRDR for each of the four reviews (forms available upon request). We want the results of our trial to be broadly applicable across a wide range of review topics, and thus, the forms include recommended common data elements (Table 2) [13, 18]. In keeping with best practices of form development [13], each form comprises predominantly pre-populated multiple-choice or numerical entry data items. We have organized the data elements into separate “tabs” in SRDR: Design Tab (study design, risk of bias), Baseline Tab (characteristics of participants by study arm at baseline), Outcomes Tab (list of outcomes reported in the article), and Results Tab (quantitative results data for the outcomes). Table 2 lists the various data elements that are contained in each tab, framed as answerable questions. Note that some data elements include multiple data items. The total number of data items varies between articles, depending upon the review topic, number of outcomes, and the amount of information available in each article. The form has a median of 121 multiple-choice or numerical entry data items (interquartile range 102 to 150, range 71 to 176).

**Table 2:** Data elements by tab in each abstraction form used in the trial

Tab	Data element
Design	Study eligibility criteria
	Number of study centers
	Region of study participant recruitment
	Start year of study participant recruitment
	End year of study participant recruitment
	End year of randomized study participant follow-up
	Length of planned (or stated) randomized study participant follow-up
	Report of a study sample size/power calculation
	Report of conduct of an intention-to-treat analysis
	Presence of a participant flow diagram in the article
	Study method to generate the random sequence
	Risk of bias related to random sequence generation
	Study method to conceal the random allocation sequence
	Risk of bias related to concealment of the random allocation sequence
	Masking (or blinding) of study participants to treatment assigned
	Masking (or blinding) of healthcare providers to treatment assigned
	Masking (or blinding) of outcome assessors to treatment assigned
	Report of “single,” “double,” or “triple” masking without clarification
	Report of absence of any masking during the study
	Sources of monetary or material support for the study
	Financial relationships for any author of the study article
	Total number of randomized study arms (or groups)
	Number of study participants randomized, by group (or arm)
	Number of study participants followed up, by group (or arm)
	Whether reasons to follow up were similar between the groups (or arms)
	How much time the abstractor spent abstracting data for the Design Tab
Baseline	Sample size at baseline, by group (or arm)
	Age at baseline, by group (arm)
	Sex at baseline, by group (arm)
	Other baseline characteristics as appropriate (e.g., body mass index), by group (arm)
Outcomes	Each outcome from a pre-defined list of outcomes with time-points specific to articles from each review
Results	For each *dichotomous outcome* at the relevant time-point:
	Number of participants analyzed, by group (arm)
	Number of participants with the outcome, by group (arm)
	Percentage of participants with the outcome, by group (arm)
	Measure of association (e.g., relative risk, odds ratio), by between arm comparison
	95% CI for the measure of association, by between arm comparison
	*P*-value for the measure of association, by between arm comparison
	For each *continuous outcome* at the relevant time-point:
	Number of participants analyzed, by group (arm)
	Mean of outcome, by group (arm)
	Standard deviation of outcome, by group (arm)
	Mean difference, by between arm comparison
	95% CI for the mean difference, by between arm comparison
	*P* value for the mean difference, by between arm comparison

The DAA trial does not include stopping rules or a Data Safety and Monitoring Board because the trial does not evaluate the safety or effectiveness of an intervention on health outcomes. We do not expect any adverse events as a result of abstracting data during this trial.

### Outcomes

The two primary outcomes for our trial are proportion of data items abstracted that constitute an error (hereafter referred to as “error rates” for simplicity) and the time taken to complete abstraction (by both abstractors, including verification/adjudication). To determine errors for Approaches A and B, we will compare the verified data to data independently abstracted and adjudicated by two investigators with extensive abstraction experience (IJS and TL), which will be considered the “answer key”. In Approach C, both abstractors edit their own abstracted data during adjudication. To determine errors for Approach C, we will use the edited data from the more experienced abstractor. We will do this by using a computer program that automatically compares the selected/entered value of a given data item to the answer key value for that data item. An error is defined as any discrepancy or difference between an entry for a data item and the answer key value for that data item. We are interested in abstraction errors resulting from omission or incorrect abstraction. If participants abstract more data items than are in the answer key, the additional data items will not be considered as errors.

The total time taken to complete abstraction for a given article is defined as the sum of the time taken for initial abstraction(s) plus subsequent verification/adjudication. To measure time, we will use three strategies. First, the study data abstraction system (i.e., SRDR) will automatically record when each abstractor logs in and out of the system, including the time spent on each tab. It is possible that this time overestimates the true time spent on the tab if the abstractor steps away from the computer. Second, as part of the Design Tab of the abstraction form in SRDR, we will ask abstractors to record the self-timed duration (in minutes) that was spent abstracting data for the Design Tab. Third, we will ask each abstractor to record the time spent (in minutes) on each step of data abstraction for each article: initial abstraction, verification, and adjudication, recorded using an online survey tool (Qualtrics^®^). We will use the automatically recorded timestamps to calculate time whenever possible, but will corroborate these data with the latter two manual strategies to assess the accuracy of our assessment of time.

To explore the impact of errors on results of meta-analyses, we also will conduct an exploratory descriptive analysis of differences among the meta-analytic estimates and 95% confidence intervals based on data derived from Approaches A, B, and C compared with those using the answer keys.

### Statistical methods

#### Statistical analysis—overview

All analyses will be conducted according to the intention-to-treat principle. We will document any protocol deviations and violations. We will compute summary error rates and time statistics for each approach, abstractor pair, review, and article, by type of question (questions in the Design Tab, Baseline Tab, or the Outcomes and Results Tabs). We expect each of our primary outcomes (error rate and time) to vary by six factors; we have included these factors as covariates in the statistical model for analyzing each of the primary outcomes. These factors include question type (design, baseline, results), abstractor pair (1 to 24), abstraction sequence (1 to 6), abstraction approach (A, B, C), article (1 to 48), and review from which articles were obtained (1 to 4). We will define errors for each data item as a binary variable (correct/incorrect) and time as a continuous variable. We will analyze error rates using logistic regression and time using linear regression. Each outcome will be analyzed in terms of the six factors using a mixed effects regression model.

#### Statistical analysis—technical details

For modeling error rates, let *p*_hijklmn_ be the probability of an error for the *h*th item of the *i*th question type abstracted by the *j*th abstractor pair following the *k*th abstraction sequence under the *l*th abstraction approach from the *m*th article obtained from the *n*th review. Then, the logistic regression model is

$$ \mathrm{logit}\ {p}_{\mathrm{hijklmn}} = a + {q}_i + {b}_l + {g}_k + {d}_n + {h}_{j(k)} + {z}_{m(n)} + {(bq)}_{li} + {(bg)}_{lk} + {(bd)}_{\ln } + \dots $$

where “…” indicates additional interaction terms one may wish to add. Question type (*q*_*i*_), abstraction approach (*b*_*l*_), and abstraction sequence (*g*_*k*_) are fixed factors; abstractor pair (*h*_*j*(*k*)_), review (*d*_*n*_), and article (*z*_*m*(*n*)_) are random factors. The key term of interest is *b*_*l*_, representing the main effect of the abstraction approach. The interaction terms (*bq*)_*li*_, (*bg*)_*lk*_, and (*bd*)_*ln*_ in the model examine whether differences between the abstraction approaches vary with type of questions asked on the abstraction form (since some questions might be easier to answer than others), abstraction sequence (which might indicate a learning effect or, more formally, a carry-over effect), and review (which might indicate a level of difficulty effect), respectively. We will check whether other factors explain differences among the abstractors (e.g., level of experience) or among the articles (e.g., better reporting). Each random effect is assumed to follow a normal distribution centered at zero with its own variance component. Correlations among items taken from the same article, for example, are explained by the common variance components. The model for the continuous time outcome has a similar structure, but utilizes a linear regression modeling the mean time with normally distributed errors. We will examine both error rates and time adjusting for type of question on the abstraction form and will also examine error rates and time for each type of question separately. We will compare automatically recorded and self-reported times, and analyze each separately.

#### Subgroup analyses

We will conduct exploratory subgroup analyses to examine whether the differences between the three abstraction approaches vary by question type, abstractor pair, abstraction sequence, article, and review, as specified in the statistical model (see the “Statistical analysis—technical details” section).

#### Missing data and sensitivity analysis

We anticipate that missing data may occur if abstractors do not finish all six articles assigned. We will make every effort to retain all abstractors and encourage complete data collection through (1) describing sufficient detail about the trial, problems caused by missing data, and the need for commitment to the trial as part of the consent process before enrollment; (2) maintaining frequent contact and sending reminders to abstractors; and (3) compensating abstractors US $250 for their time once they have completed all assignments. We will ask and report reasons for those who discontinue some or all types of participation.

In handling missing data items, we will compare the characteristics of the missing and non-missing items to determine the nature of the missing data mechanism. If data appear to be missing at random, inference can be drawn based on the observed data mixed model likelihood, as the predictor factors are all part of the design and therefore will be known. If the missing at random assumption appears invalid, we will conduct sensitivity analyses to assess robustness of findings to different missing not at random scenarios.

#### Sample size and power calculation

The purpose of our trial is to determine whether (1) use of DAA (Approach A) improves accuracy (i.e., reduces error rates) compared with Approach B, and maintains the accuracy of the usual Approach C; and (2) use of DAA improves efficiency (i.e., reduces abstraction time) compared with Approaches B and C. The adjudicated abstractions of the experienced study investigators (IJS and TL) will be used as the answer key for comparison.

The design in Table 1 includes factors related to the abstractor pair, abstraction sequence, article, and review. For determining necessary sample sizes, we make the simplifying assumption that the error rates and abstraction time per article will not depend on pair, sequence, or review. We consider this trial as having a crossover design in which each of the 48 articles is abstracted three times, once under each abstraction approach.

In our pilot study, we found that the proportion of items incorrectly abstracted by inexperienced abstractors was 24% [7]. If an experienced reviewer caught and corrected half of these errors, the error rate would be reduced to 12%; this is what we used in sample size and power calculations for Approach B. We assume that DAA-facilitated single abstraction plus verification (Approach A) would reduce the error rate relative to traditional single abstraction plus verification (Approach B), but perhaps not relative to independent dual abstraction plus adjudication (Approach C). Given the expected error rate, we want to be able to estimate the difference in error rate to within 1 or 2% in order to be able to determine which approach is meaningfully more accurate. For example, with an error rate of 12% for Approach B, we will be able to detect a statistically significant difference between Approaches A and B if Approach A’s error is less than 10%.

Using the error rate as the outcome for calculating sample size, we would expect a standard deviation for a 100-item form to be about 3% (exact if the proportion were 9%). The standard error of the difference is then *σ*(1 − *ρ*)/*N*, where *σ* is the within-unit standard deviation, *N* is the number of units (articles) receiving each sequence of crossovers (Approaches A, B, and C), and *ρ* is the correlation between two measurements on the same unit. The standard error for the difference in a crossover trial when comparing two approaches with 24 units each receiving each approach in one of two sequences, given a standard deviation per unit of 3%, ranges from 0.125%, when *ρ* = 0, to 0%, when *ρ* = 1, decreasing linearly. Thus, even if measurements are uncorrelated (i.e., *ρ* = 0), our design would be able to detect a very small difference between error rates.

Likewise, we might assume that Approach A would reduce the total time needed to abstract data relative to Approaches B and C, and we would want to estimate this within a few minutes in order to determine meaningful differences in efficiency. The same calculation shows that even with independent measurements, the standard error of the difference between the average times for two different approaches would be no more than *σ*/24, which would be less than 2 min for *σ* as large as 48 min (48 min is much longer than we would expect for the amount of data to be abstracted and the length of the articles).

### Confidentiality

We will not share trial data until the trial is completed and primary analyses are done. There are no personally identifying markers for participants in the data we collect. A file linking participant names with the assigned abstraction sequence will be accessible only to the Project Director and saved on a password-protected server maintained by JHBSPH. The server is backed up daily. We will archive trial documentation and electronic data files at the end of the trial and will retain them for at least 10 years. Because data abstracted in SRDR are associated with the abstractor’s name, we do not plan on allowing open access to the abstracted data in SRDR. However, upon request, we can make the exported de-identified abstracted data available after the trial.

### Publications and dissemination

All investigators will collaborate to disseminate trial results through manuscripts and presentations at scientific meetings. Authorship and roles in preparation of the manuscripts will be decided ahead of time and agreed by all concerned. PCORI, the funder of this trial, will convene an independent team to peer review the final report of the project and will post the finalized report on the PCORI website.

## Discussion

Current approaches to data abstraction have resulted in a large resource burden on those conducting systematic reviews; however, efforts to reduce the burden may lead to errors in abstraction. These errors could lead to inaccurate conclusions derived in reviews and, consequently, healthcare decisions that are based on faulty evidence summaries. The very foundation of evidence-based healthcare is challenged when one of its three underlying tenets (*best-available research evidence*, clinician expertise, and patient values [19]) is compromised.

### Challenges in designing the trial

The first challenge in designing this trial was to identify four reviews from which we could meet our target of identifying 12 appropriate RCTs each. Many of the reviews we selected initially could not be used because the outcomes were poorly defined in the reviews and, in some instances, meta-analyses in the reviews combined data from RCTs inappropriately. Ultimately, after ruling out various topics, we identified four suitable topics and reviews.

The second challenge was deriving the answer keys for all 48 articles. Two experienced abstractors on our investigator team (IJS and TL) abstracted data from all 48 articles. However, to be able to truly evaluate abstraction “errors” by abstractors during the trial, we needed to develop instructions and unambiguous language to clearly articulate every question and every answer option on every abstraction form for use during the trial. Ambiguous articulation of these entities on our forms could have led to information bias in a primary outcome of the DAA trial if the abstraction errors resulted not from omission or incorrect abstraction, but rather an abstractor’s misunderstanding of the abstraction requested.

To reduce the abstraction process learning curve, we are restricting the trial to individuals with at least some experience with data abstraction for systematic reviews. Another limitation is that, unlike many real-world reviews, we are not allowing resolution of abstraction discrepancies by a third abstractor.

### Strengths of the trial design

This trial has many strengths. First, it tests a novel software application (DAA) designed to make the data abstraction process more accurate and efficient. In addition, used in conjunction with a data abstraction system such as SRDR, DAA would maintain an annotated version of the data abstracted for easy access when the same or another systematic review team updates the review at a later date. Second, using a rigorous and efficient crossover design with random allocation and allocation concealment, this trial tests the effectiveness of the software application vis-à-vis two currently recommended best practice approaches to abstraction. Third, we are collaborating with patient/consumer co-investigators as the trial moves forward, in designing the trial and identifying the review topics, study articles, and outcomes for use during abstraction. Finally, the generalizability of the trial is likely to be high because of the broad eligibility criteria for abstractors from multiple locations with various types of background and levels of experience with abstraction during reviews.

In summary, current standards for data abstraction, a key step during systematic reviews, rest on a weak evidence base. Our trial represents a potentially substantial step forward in the quest to reduce errors in data abstraction. The trial will rigorously evaluate whether a novel software application (DAA) could help the systematic review community efficiently use scarce research resources. Because systematic reviews are a key component of comparative effectiveness research, our results could help improve patient care by reducing errors in the synthesis of research evidence that informs that care.

## Additional files

Additional file 1: Location of SPIRIT 2013 checklist items in this manuscript. (DOCX 47 kb)
Additional file 2: Pilot study to classify data abstractor experience with data abstraction. (DOCX 39 kb)
Additional file 3: Announcement of funding of DAA Trial by PCORI. (DOCX 486 kb)
Additional file 4: Institutional review board (IRB) approval for DAA Trial from Johns Hopkins University Bloomberg School of Public Health. (DOCX 265 kb)
Additional file 5: Institutional review board (IRB) approval for DAA Trial from Brown University. (DOCX 236 kb)

## Abbreviations

AHRQ: Agency for Healthcare Research and Quality
CCNet: Cochrane Consumer Network
CRD: Centre for Reviews and Dissemination
CUE: Consumers United for Evidence-based Healthcare
DAA: Data Abstraction Assistant
EPC: Evidence-based Practice Center
IOM: Institute of Medicine
JHBSPH: Johns Hopkins Bloomberg School of Public Health
NICHSR: National Information Center on Health Services Research and Health Care Technology
PCORI: Patient-Centered Outcomes Research Institute
PCSK-9: Proprotein convertase subtilisin/kexin type 9
RCT: Randomized controlled trial
SPIRIT: Standard Protocol Items: Recommendations for Interventional Trials
SRDR: Systematic Review Data Repository
