# Supplementary material for: Evaluating Data Abstraction Assistant, a novel software application for data abstraction during systematic reviews: protocol for a randomized controlled trial
Source: Syst Rev. 2016 Nov 22;5:196. doi: 10.1186/s13643-016-0373-7 (PMC5120497; doi:10.1186/s13643-016-0373-7)
Supplement: Additional file 2: — Pilot study to classify data abstractor experience with data abstraction. (DOCX 39 kb) [file 13643_2016_373_MOESM2_ESM.docx]

Additional file **2: Pilot study to classify data abstractor experience with data abstraction**

We conducted a pilot study to identify characteristics that best classify an individual’s level of experience in performing data abstraction for systematic reviews.

We surveyed faculty, staff, and students at two schools of public health (Brown and JHBSPH), two evidence-based practice centers (EPCs) (Johns Hopkins EPC and Brown EPC), and Cochrane United States. Respondents were eligible if they had abstracted data from at least one study for a systematic review. We asked questions on respondent’s current status (faculty, staff, student, other), number of articles abstracted, number of systematic reviews published, and self-rated level of experience with data abstraction. Masked to their responses, we labeled each respondent as either a *more* experienced or *less* experienced data abstractor, based on how we would have considered their experience in a real-life systematic review. We used our classification as the reference, and calculated the sensitivity and specificity of using 15 pre-defined items (or combination of items) and thresholds in classifying data abstractor experience. We considered the items/combination of items with the highest total of sensitivity and specificity as having the best accuracy.

We included 45 participants; 23 were classified as *less* experienced and 22 as *more* experienced data abstractors. The item on having published three or more vs. two or fewer systematic reviews had the best accuracy (sensitivity=0.73 and specificity=0.74) (see blue highlights in following table). Adding the item of number of articles abstracted, either dichotomized at 10 or more vs. fewer than 10 articles or dichotomized at 20 or more vs. fewer than 20 articles (see grey highlights in following table), did not improve the accuracy provided by the item of having published three or more reviews alone.

We therefore selected the number of reviews published, dichotomized at three or more vs. two or fewer, as the criterion for classifying abstractors as “more” or “less” experienced with abstraction, respectively.

Measures of accuracy of various questions and combinations of questions (using various cutoffs) for classifying data abstractor experience

| **Accuracy measure** | **Estimate** | **95% CI LL** | **95% CI UL** | **Accuracy measure** | **Estimate** | **95% CI LL** | **95% CI UL** |
| --- | --- | --- | --- | --- | --- | --- | --- |
| *Abstracted from ≥10 articles AND*  *Published ≥1 systematic review* | | | | *Abstracted ≥20 articles AND*  *Self-rating as “very experienced”* | | | |
| Sensitivity | 0.91 | 0.83 | 0.99 | Sensitivity | 0.50 | 0.35 | 0.65 |
| Specificity | 0.43 | 0.29 | 0.58 | Specificity | 0.78 | 0.66 | 0.90 |
| *Abstracted from ≥ 10 articles AND*  *Published ≥3 systematic reviews* | | | | *Abstracted ≥20 articles AND*  *Published ≥1 systematic review AND*  *Self-rating as “very experienced”* | | | |
| Sensitivity | 0.73 | 0.60 | 0.86 | Sensitivity | 0.45 | 0.31 | 0.60 |
| Specificity | 0.74 | 0.61 | 0.87 | Specificity | 0.83 | 0.72 | 0.94 |
| *Abstracted from ≥20 articles AND*  *Published ≥1 systematic review* | | | | *Published at least 3 systematic reviews* | | | |
| Sensitivity | 0.86 | 0.76 | 0.96 | Sensitivity | 0.73 | 0.60 | 0.86 |
| Specificity | 0.57 | 0.42 | 0.71 | Specificity | 0.74 | 0.61 | 0.87 |
| *Abstracted from ≥20 articles AND*  *Published ≥3 systematic reviews* | | | | *Abstracted ≥20 articles OR*  *Published ≥3 systematic reviews* | | | |
| Sensitivity | 0.73 | 0.60 | 0.86 | Sensitivity | 0.95 | 0.89 | 1.02 |
| Specificity | 0.74 | 0.61 | 0.87 | Specificity | 0.39 | 0.25 | 0.53 |
| *Abstracted ≥10 articles AND*  *Self-rating as “very experienced”* | | | | *Current status Faculty or PhD student* | | | |
| Sensitivity | 0.50 | 0.35 | 0.65 | Sensitivity | 0.68 | 0.55 | 0.82 |
| Specificity | 0.78 | 0.66 | 0.90 | Specificity | 0.74 | 0.61 | 0.87 |
| *Abstracted ≥10 articles* | | | | *Published ≥3 systematic reviews OR*  *Current status Faculty or PhD Student* | | | |
| Sensitivity | 1.00 | 1.00 | 1.00 | Sensitivity | 0.91 | 0.83 | 0.99 |
| Specificity | 0.22 | 0.10 | 0.34 | Specificity | 0.48 | 0.33 | 0.62 |
| *Published ≥3 systematic reviews AND*  *Self-rating as “very experienced”* | | | | *Published ≥3 systematic reviews AND*  *Current status Faculty or PhD Student* | | | |
| Sensitivity | 0.45 | 0.31 | 0.60 | Sensitivity | 0.45 | 0.31 | 0.60 |
| Specificity | 0.87 | 0.77 | 0.97 | Specificity | 1.00 | 1.00 | 1.00 |
| *Abstracted at least 20 articles* | | | |  | | | |
| Sensitivity | 0.95 | 0.89 | 1.02 |  |  |  |  |
| Specificity | 0.39 | 0.25 | 0.53 |  |  |  |  |
