# Supplementary material for: Evaluating Data Abstraction Assistant, a novel software application for data abstraction during systematic reviews: protocol for a randomized controlled trial
Source: Syst Rev. 2016 Nov 22;5:196. doi: 10.1186/s13643-016-0373-7 (PMC5120497; doi:10.1186/s13643-016-0373-7)
Supplement: Additional file 3: — Announcement of funding of DAA Trial by PCORI. (DOCX 486 kb) [file 13643_2016_373_MOESM3_ESM.docx]

Additional file **3: Announcement of funding of DAA Trial by PCORI**

**(Screenshot of** [**http://www.pcori.org/research-results/2014/develop-test-and-disseminate-new-technology-modernize-data-abstraction**](http://www.pcori.org/research-results/2014/develop-test-and-disseminate-new-technology-modernize-data-abstraction)**; Screenshot captured on September 6, 2016)**

**
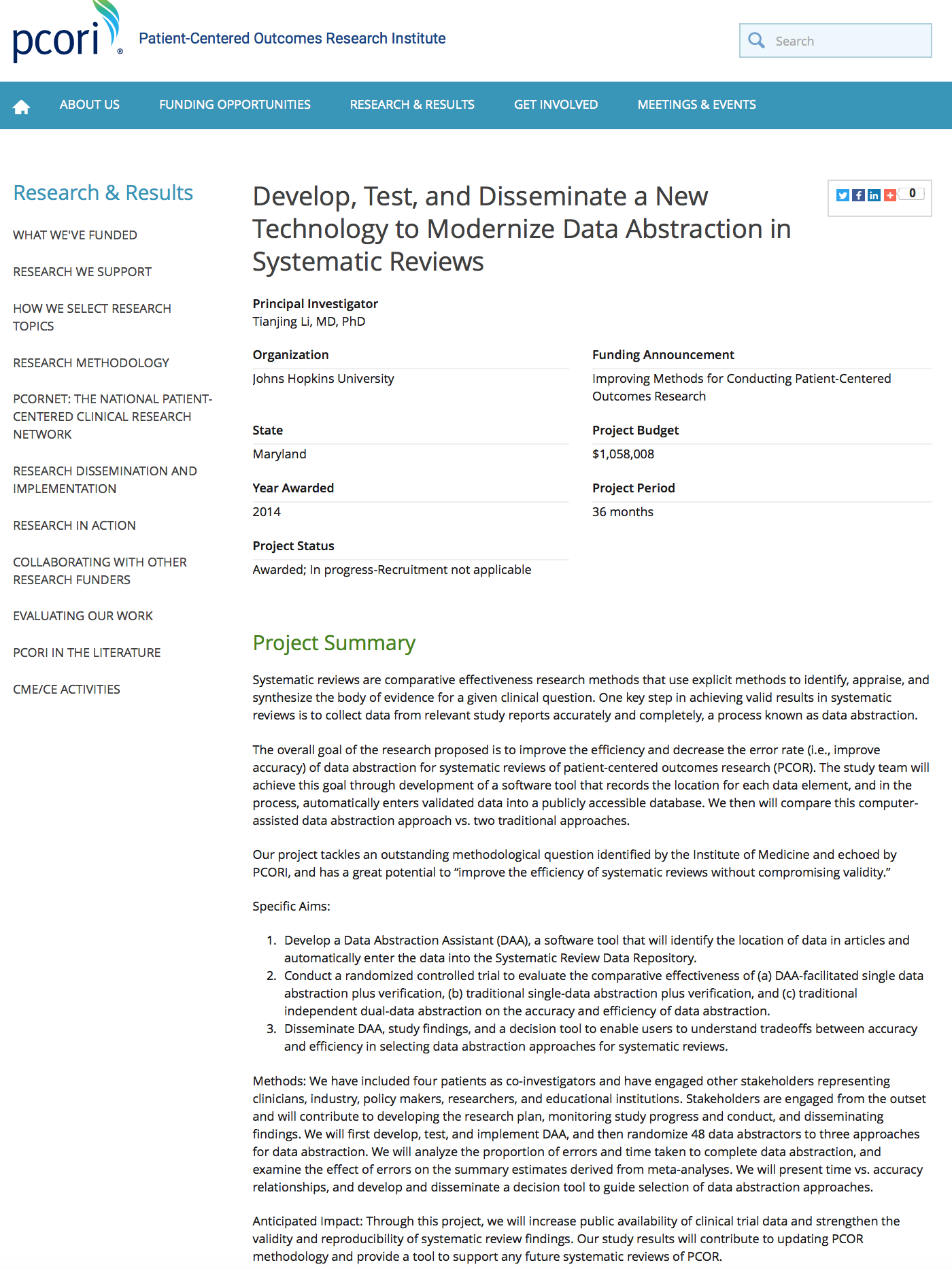
**
