# Supplementary material for: Evaluating Data Abstraction Assistant, a novel software application for data abstraction during systematic reviews: protocol for a randomized controlled trial
Source: Syst Rev. 2016 Nov 22;5:196. doi: 10.1186/s13643-016-0373-7 (PMC5120497; doi:10.1186/s13643-016-0373-7)
Supplement: Additional file 4: — Institutional review board (IRB) approval for DAA Trial from Johns Hopkins University Bloomberg School of Public Health. (DOCX 265 kb) [file 13643_2016_373_MOESM4_ESM.docx]

Additional file **4: Institutional Review Board (IRB) approval for DAA Trial from Johns Hopkins University Bloomberg School of Public Health**

**
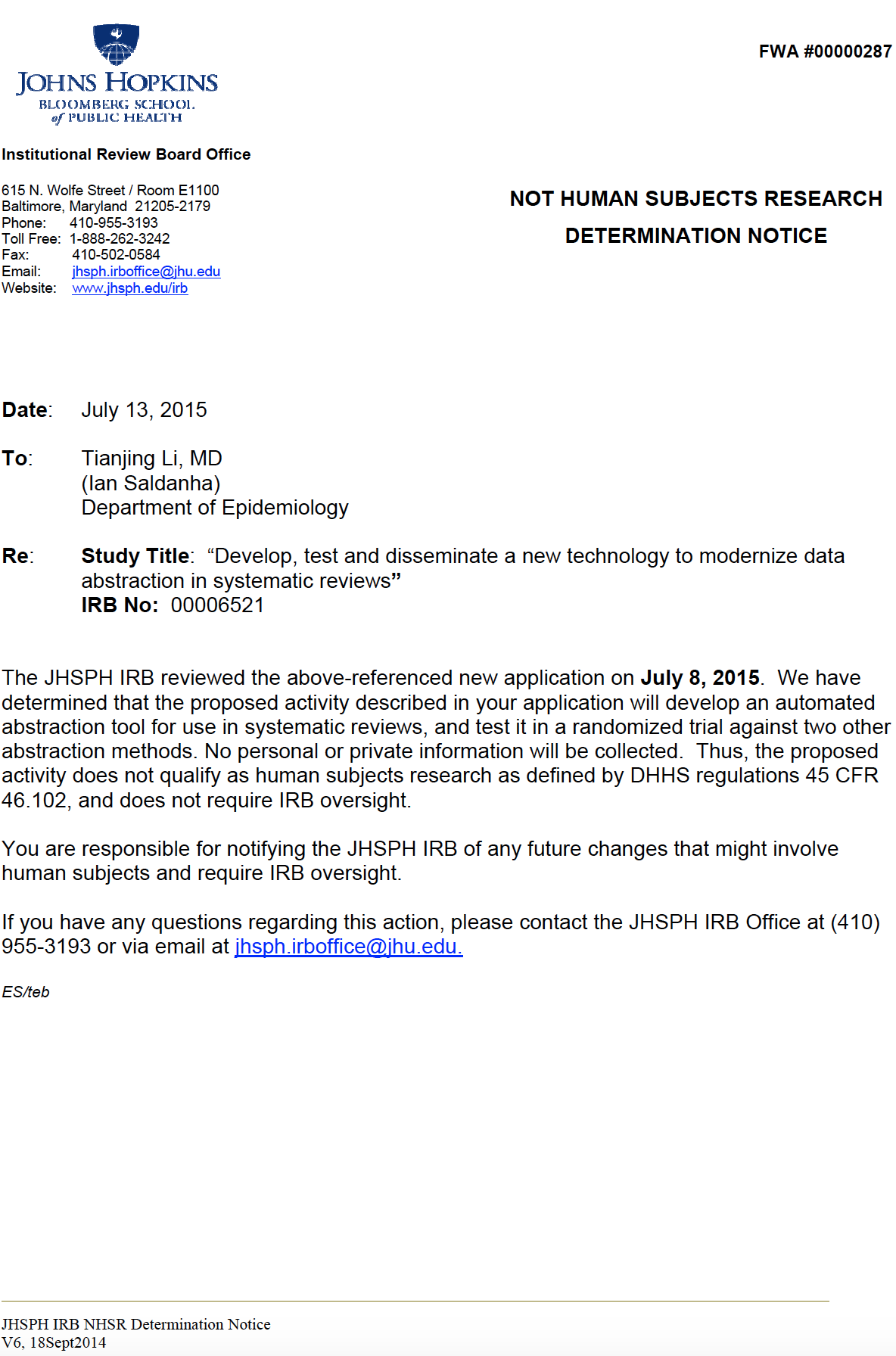
**
