# Supplementary figures and images for: Evaluating Data Abstraction Assistant, a novel software application for data abstraction during systematic reviews: protocol for a randomized controlled trial
Source: Syst Rev. 2016 Nov 22;5:196. doi: 10.1186/s13643-016-0373-7 (PMC5120497; doi:10.1186/s13643-016-0373-7)

Additional file **5: Institutional Review Board (IRB) approval for DAA Trial from Brown University**

**
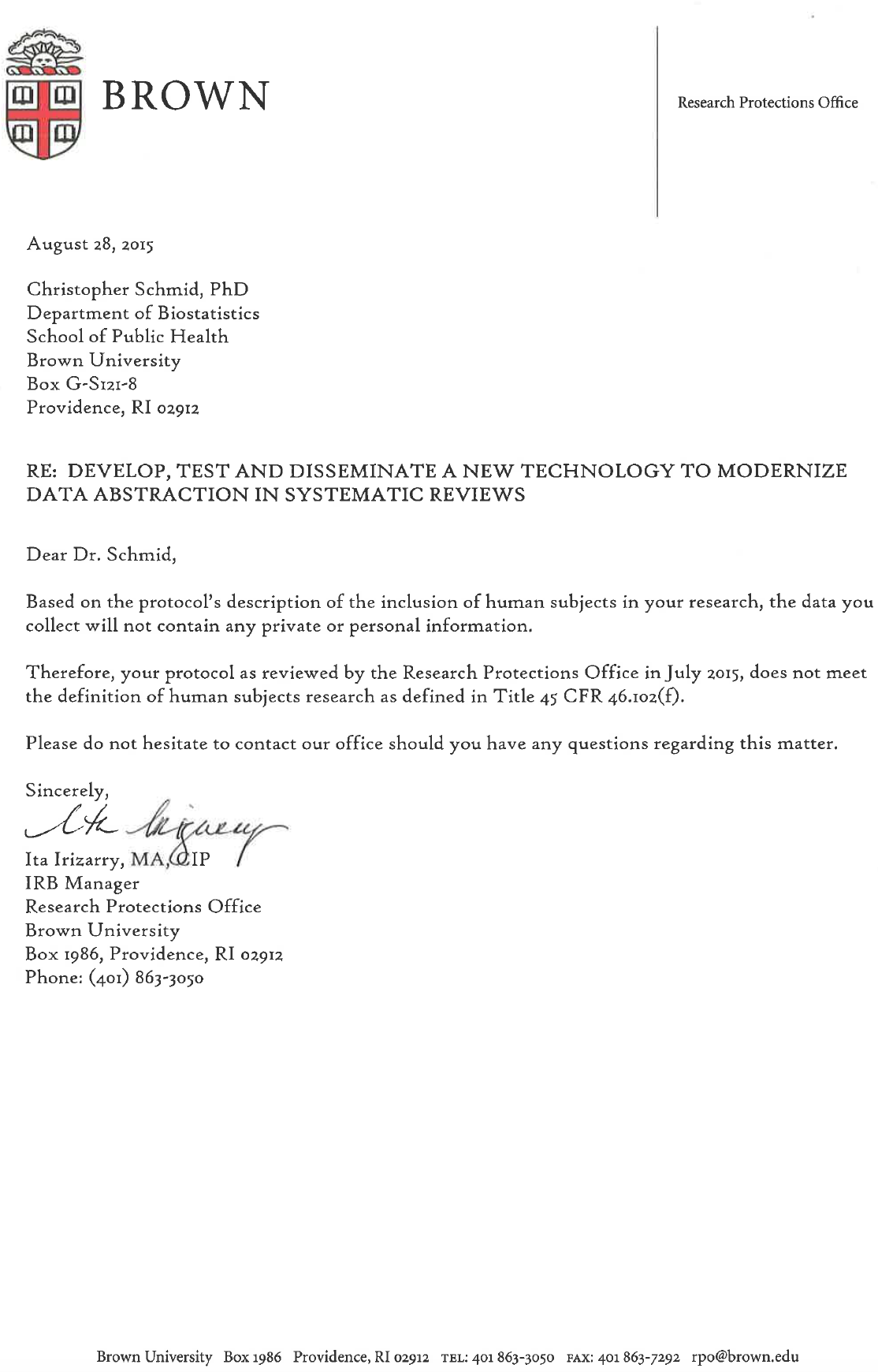
**

Supplement: Additional file 5: — Institutional review board (IRB) approval for DAA Trial from Brown University. (DOCX 236 kb) [file 13643_2016_373_MOESM5_ESM.docx]
